# Texas State Medical Association—Thirty-first Annual Meeting, San Antonio, Texas, April 25 to 28, 1899

**Published:** 1899-05

**Authors:** 


### Society Notes.

### Texas State Medical Association—Thirty-first Annual Meeting, San Antonio, Texas, April 25 to 28, 1899.

### REPORT OF COMMITTEE ON STATE BOARD OF HEALTH.

Dr. R. H. Harrison, Chairman, read the following report, which was adopted:

"Your committee charged with the duty of formulating a law providing for the creation of a State Board of Health and Vital Statistics, and urging the passage of the same by the Twenty-sixth Legislature of the State, entered upon the discharge of its duty confident that the importance of the subject and the great benefits to be conferred by a judicious work of that character, would insure a successful result. The prospective change of administration added greatly to this confidence, but we regret to say that our anticipations have not been realized.

"Soon after the organization of your committee, a convention of local health officers and municipal authorities was called, at the instance of and through the agency of the mayor and aldermen of the city of Galveston, which met in the city of Houston and afforded invaluable aid in directing its subsequent labors.

"The members of your committee were residents of sections of the State more or less remote from each other made satisfactory conference by them difficult, and on some occasions impracticable. It succeeded finally, however, in formulating a satisfactory bill, clear and comprehensive, covering the entire ground contemplated by the resolution providing for its appointment. This bill was introduced in the higher branch of our Legislature by the Honorable A. B. Kerr on the 10th day of March last, and referred by the body on account of some penalties imposed for violation of its text, to 'Judiciary Committee No. 2,' where it has lain without report ever since. It is to be regretted that this bill could not have been committed to the Senate's Committee on Public Health, whose qualifications to appreciate its importance are, presumably, far greater than those of the committee to which it was referred.

"Repeated efforts have been made by your committee to secure a report from the legislative committee on this subject, but without avail. Legislative indifference to this important subject is no less remarkable than it is reprehensible. The lives and the property of the best men in the State are placed in jeopardy by it; the health and comfort of those that are nearest and dearest to every citizen of the commonwealth is imperiled. And is it too much to say that the parties whose duty it is to provide laws for the protection of life and property make themselves the accomplices of homicide in every preventable death that occurs from such neglect?

"In view of all circumstances, your committee would recommend the adoption by the Association of a memorial to the Governor and members of the Legislature, inviting their attention to the necessity for speedy action in the premises. Your committee submit herewith an outline of such a memorial, and feel assured that members of the Association will make such use of it as will result in the greatest benefit to the State.

"If all the individual members of the Association should append their names to a memorial of this Association and let it go at once to the Governor and the members of the Legislature, it would probably be the most effective method we could adopt to secure results."

### MEMORIAL TO THE GOVERNOR AND LEGISLATURE.

Asking for sanitary legislation in the interest of the public health.

Dr. Harrison then read the following memorial:

*"To His Excellency the Governor, and to the Honorable the Members of the Twenty-sixth Legislature:*

"Your memorialists respectfully solicit your attention to the apparent and palpable necessity for the revision of our State health laws. The preservation of life and the protection of property were unquestionably the primary incentives to the formation of organized government. The progress of civilization has introduced new elements, imposing additional obligations upon the statesmen of the nation.

"That these new elements and additional obligations have taken precedence of the primary obligations for which governments were formed, seems to be a self-evident proposition. Yet the State spends annually half a million dollars (more or less) in the prosecution of criminals, ostensibly for the protection of human life. The futility of that expenditure is made so obvious by reference to the records of our courts, that we can only wonder. But this is foreign to the purpose of this paper.

"The constitution of the State provided for the creation of a State Board of Health and Vital Statistics (Sec. 23 of Article 16 of the Constitution of 1876). Former Legislatures have ignored this provision of the State's organic law, substituting a statute for the appointment, merely, of a State Health Officer. This 'State Health Officer' is charged with the duty of administering quarantine against imported diseases, and the exercise of a general supervision over the health of the State, especially it is made his duty to make all necessary investigations and inquiry as to the adulteration of food and drugs.

"That the different incumbents of this office have invariably restricted their duties to an effort to protect the State against the introduction of yellow fever will, we think, be fully shown by reference to the records of that office. Other infectious and contagious diseases have been almost, if not entirely, ignored—as has also that part of the statute referring to the examination of foods and medicines; while it is notorious that counterfeits of both (particularly lard and butter foods) and large numbers of medicines are constantly on the markets of the State, to the great detriment of the public health and jeopardy of human life (see Article 4317 under Title 91, Sayles' Digest).

"But your memorialists desire particularly to call your attention to the fact that 'State Medicine' is now a prominent factor in the more advanced statesmanship of all the civilized nations of the earth. England, France, Germany, and even semi-barbarous Hungary, have all provided effective organizations for the administration of sanitary laws for the benefit of their people. Your memorialists have been unable to secure a copy of the health laws of the Republic of Mexico, but it is understood that the enlightened government of that young nation has recently taken an advanced position on the subject of public sanitation. Indeed, 'State Medicine,' 'public hygiene,' or some other title of similar significance, constitutes a special branch of teaching in most of the prominent medical schools of the world.

"Massachusetts was the first of the United States to provide a Board of Health for the protection of the lives and property of her citizens. Massachusetts was the first of the United States to suffer from yellow fever. More than two hundred years ago, when the city of Boston was a village, as compared with the present population, yellow fever swept 2600 human lives out of existence. Subsequent visitations were only a little less fatal. Effective sanitary measures have long since rendered them exempt. Philadelphia and New York were the next places visited by the pestilential scourge. They, too, are now considered exempt, as is also Baltimore—the latter standing pre-eminent for its cleanliness.

"Thirty States of the Union, within the knowledge of your memorialists, have availed themselves of the protection of life and property afforded by State Boards of Health. Organizations of this kind in many States, it is true, are imperfect and fall far short of the accomplishment of the purposes for which they were instituted. In others, however, as, for instance, the States of Massachusetts, Pennsylvania, New York, Ohio, Michigan, Tennessee, Florida and Alabama, have organizations of supreme excellence, the benefits of which are beyond computation. We do not mention the Boards of Health of Mississippi and Louisiana, because the results of their labors, while appreciated by your memorialists, are less palpable to the popular mind.

"We may refer, however, to the comparatively new State of Colorado, whose Board of Health and Vital Statistics have contributed more (through their annual reports) to the material interests of that commonwealth than all her mineral deposits. Even the Territory of Oklahoma, profiting by the prosperity secured to her neighbor, through the agency of its Board of Health and Vital Statistics, has been prompt to avail herself of a similar organization, and is now reaping great benefits therefrom, in the acquisition of desirable citizens.

"The comparatively healthy condition of our State, and the apparently low death rate in its population, may to some extent excuse former Legislatures for negligence on this important subject; but the recent widespread prevalence of smallpox, with all of its loathsomeness and fatalities, the no less widespread prevalence of cerebro-spinal meningitis, with the fearful death rate that attends its progress, seem to demand of the present Legislature prompt and efficient action on this subject.

"That our present health laws are wholly insufficient for the protection to which people are entitled, is made conspicuously manifest by the results of the epidemic now prevalent in Laredo, and the extraordinary measures adopted to abate it. That this foul disease has been distributed from that point, or some other, despite the vigilance of the present Health Officer, over a very large minority of the counties of the State, is apparent from constant reports in the daily press during the last three or four months.

"Your memorialists made an effort to secure accurate information as to the number of counties in which it has appeared, and an application to the State Health Officer for information on this subject was met with the response that his was merely a quarantine office, and kept no record of the character requested.

"The damage which has accrued to the State from this pestilential visitation is beyond computation. Its pecuniary cost to the people alone has been enormous, to say nothing of the destruction of life, more or less valuable, and the maiming and disfiguring of individuals. Add to this the bad fame acquired by the State on account of the widespread distribution of the disease, and you can imagine, approximately, the extent of the damage.

"While the territory over which this meningitis has prevailed has probably been equally as great as that occupied by the smallpox, the number of cases has been much smaller, though fatal results have been far greater, and yet our legislators sit supinely in their seats and take no steps to secure protection against pestilential visitations, or discover the cause, etc.

"It is needless to call your attention to that class of diseases now universally recognized as preventable—typhoid fever, measles, scarletina, diphtheria, etc., which are to many sections of the State endemic. They have been with us always, and the mass of the people are so accustomed to them that they attract but little attention, notwithstanding more people die from these several diseases every day than have ever died on any day from yellow fever.

"The functions of a Board of Health, such as the State ought to provide, may be amply summed up to comprehend a general supervision of the health of the State, whenever and wherever it may be made effective. Its ramifications should extend into, at least, all the most populous counties of the State, with tributary organizations also in the large cities. Thus constituted, it should be charged with the duty of investigating the causes, character and means of arresting, or preventing, all epidemic and endemic diseases with which the different sections of the State may be affected. Climatic observations should constitute an important part of their duty, as well as the influence of temperature, rainfall, prevailing wind, etc., upon the public health. The application of the principles of sanitary science should be inculcated by it, whenever and wherever the same could be available. In addition to these duties, provision should be made for the preservation of the vital statistics of the State, without which her material interests must frequently suffer, as every lawyer knows, to say nothing of the fair fame as a health resort.

"A committee of the State Medical Association formulated a law of this character, and it was introduced in the Senate by the Hon. A. B. Kerr. In consequence of some penalties involved, it was referred to the Judiciary Committee No. 2, instead of the Committee on Public Health, who would have been more competent to appreciate its importance. It has been in the hands of this committee for many weeks without report.

"Legislative wisdom has heretofore provided 'a board of health' for the protection of the lives and comfort of our cattle in the sanitary commission 'for live stock,' thus recognizing as a fact that the life of a cow is of more importance to the State of Texas than a human being. This policy, however, is in keeping with the finding of our courts, which rarely punishes a murderer, while the theft of a yearling is generally given a term in the penitentiary."

The report of the committee was adopted, and the following committee was appointed to convey the memorial to Governor Sayers: Dr. W. M. Cunningham, of Bastrop; Dr. R. H. Harrison, of Columbus; Dr. M. M. Smith, of Austin; Dr. James H. Bell, of San Antonio, and Dr. A. N. Denton, of Austin.

